# Cerebral Vessels: An Overview of Anatomy, Physiology, and Role in the Drainage of Fluids and Solutes

**DOI:** 10.3389/fneur.2020.611485

**Published:** 2021-01-13

**Authors:** Nivedita Agarwal, Roxana Octavia Carare

**Affiliations:** ^1^Hospital S. Maria del Carmine, Azienda Provinciale per i Servizi Sanitari, Rovereto, Italy; ^2^Laboratory of Functional Neuroimaging, Center for Mind/Brain Sciences, University of Trento, Trento, Italy; ^3^Faculty of Medicine, University of Southampton, Southampton, United Kingdom

**Keywords:** cerebral vessel, glymphatic, intramural periarterial drainage, small vessel disease, neurodegeneration, perivascular space

## Abstract

The cerebral vasculature is made up of highly specialized structures that assure constant brain perfusion necessary to meet the very high demand for oxygen and glucose by neurons and glial cells. A dense, redundant network of arteries is spread over the entire pial surface from which penetrating arteries dive into the cortex to reach the neurovascular units. Besides providing blood to the brain parenchyma, cerebral arteries are key in the drainage of interstitial fluid (ISF) and solutes such as amyloid-beta. This occurs along the basement membranes surrounding vascular smooth muscle cells, toward leptomeningeal arteries and deep cervical lymph nodes. The dense microvasculature is made up of fine capillaries. Capillary walls contain pericytes that have contractile properties and are lined by a highly specialized blood–brain barrier that regulates the entry of solutes and ions and maintains the integrity of the composition of ISF. They are also important for the production of ISF. Capillaries drain into venules that course centrifugally toward the cortex to reach cortical veins and empty into dural venous sinuses. The walls of the venous sinuses are also home to meningeal lymphatic vessels that support the drainage of cerebrospinal fluid, although such pathways are still poorly understood. Damage to macro- and microvasculature will compromise cerebral perfusion, hamper the highly synchronized movement of neurofluids, and affect the drainage of waste products leading to neuronal and glial degeneration. This review will present vascular anatomy, their role in fluid dynamics, and a summary of how their dysfunction can lead to neurodegeneration.

## Introduction

Damage to cerebral vasculature and reduction in cerebral perfusion initiate a cascade of events that rapidly leads to disturbed cellular homeostasis and death of neurons and glial cells (1). The cerebral arterial network of vessels is unique in its anatomy, and its flow dynamics is inextricably intertwined with those of other fluids such as venous blood, cerebrospinal fluid (CSF), and the interstitial fluid (ISF) (2, 3). Emerging evidence regarding the role of cerebral vasculature in the drainage of solutes and fluids adds to the complexity of the overall interaction with neurofluids.

The arteries of the brain have a dual function: to supply oxygenated blood to neurons and glia and to drain ISF. Neurons and glial cells are constantly “at work,” even during rest, and this very high demand for oxygen and glucose requires a steady supply of oxygenated blood. Histological and tracer studies reveal the intricate relationship of cortical arteries with meningeal sheaths and the constitution of the perivascular compartment and spaces that provide a pathway for inflow and outflow of ISF (4–6). Cerebral capillaries are considered important sites of CSF and ISF production and absorption. Capillaries drain into venules that are hierarchically organized and run centrifugally toward the cortex. All venous drainage occurs through dural venous sinuses that drain toward the neck veins. The walls of dural venous sinuses are also home to meningeal lymphatic vessels (7, 8), with a role in the drainage of CSF. In this review, a brief overview of the current evidence for the anatomy and function of vessels in the brain will be provided, followed by a summary of mechanisms of interaction of what we term “neurofluids”: blood, CSF, and ISF (2). A disruption of such mechanisms will trigger a series of pathological events such as microvascular injury, failure of ISF drainage, local deposition of amyloid-beta as cerebral amyloid angiopathy (CAA), focal ischemia, and demyelination.

### Arterial and Capillary Systems

The brain parenchyma is supplied by two internal carotid arteries (ICAs) and two vertebral arteries. The ICA enters the skull-base through the carotid canal, located in the petrous portion of the temporal lobe. It pierces through the dura mater at the level of the cavernous sinus and bifurcates within the subarachnoid space (SAS) into middle cerebral arteries and anterior cerebral arteries. The ICA carries ~80% of the total blood to the brain. The vertebral arteries enter the vertebral foramina at the level of C6; they exit out of C1 foramina, loop around the posterior arch of the atlas as they enter the foramen magna, and lies on the ventral surface of the brain stem to form the basilar artery (BA). The BA terminates into two posterior cerebral arteries. The anterior (ICA and its branches) and the posterior circulation (vertebral arteries and its branches) arteries come together at the base of the skull to form the circle of Willis that lies in the cisternal space (9).

**Figure 1:**
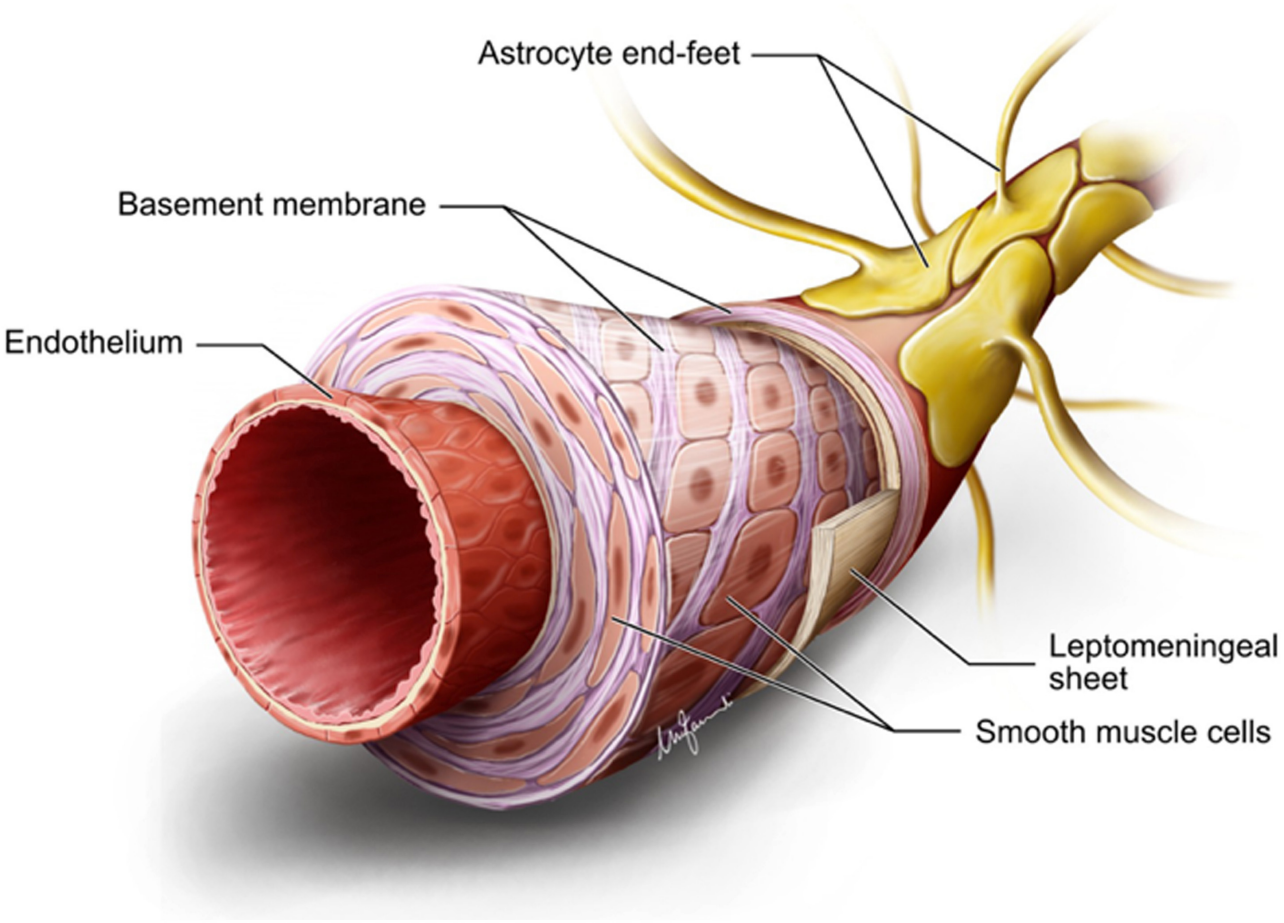
Diagrammatic summary of the structure of an arteriole in the gray matter. Endothelium hosts the blood–brain barrier. There are several layers of smooth muscle cells separated by basement membranes. Adventitial leptomeningeal sheath has its own basement membrane that fuses with the basement membrane of astrocyte end feet to form a perivascular compartment or perivascular space. Diagram drawn by Marco Fanuli.

A rich, anastomotic network of leptomeningeal arteries spreads over the pial surface from which numerous branches (arterioles) sprout out and pierce the glia limitans to dive into the cortex at approximately right angles to it. From a structural point of view, both pial arteries and penetrating arterioles lack an external elastic lamina, but leptomeningeal arteries retain an internal elastic lamina (10). The gray matter (GM) has a larger number of arterioles with respect to those in the white matter (WM) with a ratio of 8:1, which is proportionate to the elevated energy demand of the more cellular GM (11, 12). Penetrating arterioles are completely encased by a sheet of pia mater, which reflects off the cortical surface, separating them from the surrounding SAS and the brain parenchyma (4) (Figure 1). However, around the perivascular compartment of the arterioles in the WM, there are two such sheaths, creating a potential space for the accumulation of edematous fluid (13). At the capillary level, direct observations under the electron microscope in a variety of species reveal that the basement membrane of the pial sheath and the basement membranes of the astrocytes (glia limitans) fuse together to create a perivascular compartment, or periarterial space, filled with an extracellular matrix (ECM), which is not continuous with the SAS (4, 14) and referred to as the “perivascular space” (PVS) (Figure 1). Indeed, PVSs, or more appropriately the periarterial spaces, are not visible within the cortical GM even under pathological conditions, whereas they are seen in the WM both in histological specimens and on neuroimaging (13, 15). Changes in the walls of capillaries and arterioles associated with aging, hypertension, or diabetes mellitus lead to small vessel disease (SVD) and vascular dementia (16, 17).

Pial surface arterial networks are richly innervated by sympathetic nerves from the superior cervical ganglion, sphenopalatine, otic, and trigeminal ganglia that release several neurotransmitters and neuromodulators such as a vasoactive intestinal peptide, nitric oxide synthase, acetylcholine, norepinephrine, and substance P (18). This innervation, also termed “extrinsic” innervation, ends in the precapillary segment and, more precisely, where the PVS terminates. The extrinsic innervation is primarily responsible for a prompt myogenic response to temporary pressure differences. According to Poiseuille's law, a change in radius directly affects resistance to flow to the fourth power, thereby modulating blood flow instantly and efficaciously (19). Intraparenchymal arterioles are innervated by nerves arising from the nuclei of basal forebrain such as the locus coeruleus, nucleus basalis of Meynert, and raphe nuclei in the brain stem that release norepinephrine, acetylcholine, and 5-hydroxythyrosine as well as other neuropeptides either directly to the walls of capillaries or indirectly via local interneurons and astrocytes (18, 20). Such nerve endings are likely to control the intrinsic spontaneous contractile activity of vascular smooth muscle cells (VSMCs) in the tunica media, also termed “vasomotion.” Vasomotor oscillations form the basis of ultra-slow 0.1-Hz wave activity in the microvasculature independent of neuronal activity (21, 22).

A dense capillary anastomotic network characterizes the GM and varies with its depth (23). Approximately 50–60% of total blood volume is within the capillaries (23). Capillary walls are made up of a single layer of endothelial cells, pericytes, and a basal lamina made up of collagen type IV, heparan sulfate proteoglycans, laminin, fibronectin, and other ECM proteins, in various proportions and with different isoforms depending on the type of vessel (24–26). The endothelial cells are bound together by tight junction proteins such as claudins and occludins, creating a highly regulated blood–brain barrier (BBB) that restricts transcellular flux of ions and hydrophilic solutes, shielding the internal parenchymal milieu from even the slightest fluctuations in the osmolarity of surrounding tissues and blood plasma (27, 28). The endothelium contains a spectrum of receptors essential for the entry and efflux of peptides, such as low-density lipoprotein related protein-1 or adenosine triphosphate-binding cassette transporters, which are essential for the efflux of soluble amyloid-beta from the brain parenchyma (29). The abluminal surface of the capillaries is continuous with astrocytic end feet (or glia limitans), containing aquaporin-4 (AQP-4) water channels.

### Venous System

The parenchymal microvasculature drains deoxygenated blood centrifugally from the ventricular ependymal surface toward the cortical pial surface, via medullary venules and veins arranged hierarchically and centrifugally from the ventricular ependymal wall toward the cortex (30). Large cortical bridging veins, such as the vein of Labbè and the Trolard vein, empty into the superficial dural venous sinuses (31). The superior sagittal sinus subdivides into right and left transverse sinus and continues directly via sigmoid sinuses into the internal jugular veins, extracranial neck vessels, and the intra- and extra-spinal venous plexi, conveying deoxygenated blood to the right atrium (32, 33). Deep internal veins form the inferior sagittal sinus, the vein of Galen, and the straight sinus to drain into the superior sagittal sinus posteriorly. Anterior venous drainage occurs through the cavernous sinus, sphenopetrosal sinuses, and sigmoid sinuses. Several anatomical variations exist, and veins can vary in number, size, symmetry across hemispheres, and their extracranial venous drainage patterns, adding to the complexity of the cerebral venous system. It is important to note that dural venous sinuses are valveless, making cephalad retrograde flow possible in cases of obstruction to downward flow (34).

Surrounding each parenchymal arteriole are eight venules (5). Venules typically have a larger lumen area and a thinner vessel wall with respect to arterioles (35). Exiting venules in the cortex are surrounded by an incomplete layer of pia mater (4). Paravenous spaces communicate with the SAS directly. The first reports of the presence of lymphatic vessels in the dura mater were reported in 1787, whereas histologic evidence of their existence was provided much later (36). More recently, lymphatic channels were described lining the dural venous sinuses that appear to be additional routes for the drainage of fluids and cells toward the deep cervical lymph nodes (7, 8). Lymphatic channels are also found in the cribriform plate that drains fluids, cells, and solutes via nasal lymphatics toward the superficial cervical lymph nodes (37).

### Production and Drainage of Cerebrospinal Fluid and Interstitial Fluid

Our classic understanding of CSF fluid production and absorption is being challenged, as new evidence suggests that CSF production also occurs at other sites such as the capillary endothelial surface, as formulated by the Bulat–Klarica–Orešković hypothesis (38). Almost 80% of CSF is secreted by fenestrated capillaries in the choroid plexi at a rate of ~0.3–0.4 ml/min for a total production of 430–580 ml daily. CSF secretion across the blood–CSF barrier depends on hydrostatic and osmolarity gradients that exist between the plasma and the intraventricular CSF fluids. CSF comprises 99% water, some ions, and negligible quantities of proteins and glucose. Arachnoid granulations found in the dural venous sinuses are traditionally recognized to play a prime role in CSF reabsorption. However, the contemporary presence of the meninx *primitiva* and the lack of arachnoid granulations in the fetus suggests that there must be alternative routes for its absorption (37, 39).

There are multiple sources of ISF production, such as filtration across the capillaries via the development of hydrostatic and osmotic pressures across the endothelium, secretion through choroid plexi, and cellular metabolism (40, 41). ISF fills the extracellular space (ECS) or interstitial space. This space contains an ECM made up of glycosaminoglycans, glycoproteins (e.g., laminins, collagen, chondroitin, fibronectin) and proteoglycans (e.g., hyaluronic acid, heparan sulfate). Such an environment determines a negatively charged ambient necessary for cellular communication, volume transmission, immunosurveillance, and a binding capacity for solutes to be transported around brain areas. ECS occupies ~15–20% of the total brain volume, and this volume can change in physiologic and pathologic conditions such as sleep, under anesthesia, and stroke (42–45). ISF is also the primary fluid medium for waste removal; however, the presence of BBB notably restricts the movement of proteins across the capillaries, which suggested that there must be alternative pathways. Bulk flow of ISF through the brain parenchyma was proposed as a route to flush out waste products and fluids toward the ventricular ependymal walls (46). In the past decade, multiple waste clearance pathways have been characterized in the brain: the glymphatic pathway, intramural periarterial drainage pathway (IPAD), flow along cranial nerves, and meningeal lymphatics along the dural venous sinuses (6, 39, 47), still extensively debated (48, 49). The glymphatic system proposes that CSF from the SAS recycles along the para-arterial spaces and enters the brain tissue via astrocytic AQP-4 water channels. CSF intermingles with ISF, which flows toward paravenous spaces via bulk flow, thus flushing out fluids and solutes from the brain (50, 51). However, diffusion rather than bulk flow may be the likely principal mechanism for flow with an unclear role for AQP-4 channels (40, 52–55). Also, the mechanism of unidirectional CSF flow along intraparenchymal para-arterial spaces remains debatable, as arterial pulsations alone do not determine such flow (56). Furthermore, the glymphatic hypothesis does not explain why in CAA, the deposition of proteins occurs in the tunica media of arterioles and arteries, spreading to occupy the whole of the arterial wall and rarely involves veins (57–59).

On the other hand, tracer injection studies in animal brains have unequivocally demonstrated that one important route for ISF and solute removal is the IPAD. For decades, perivascular compartments have been considered to play a fundamental role in the removal of waste products (36, 60, 61). According to this mechanism, fluids and waste products flow within the basement membranes of arterioles and arteries in the opposite direction to arterial blood flow within their lumen and is primarily driven by vasomotion (62–65). The ultraslow frequency oscillation (<0.1 Hz) appears to be critical to the clearance of solutes. Electrophysiologically observed slow-wave oscillations characteristic of sleep are intricately associated with large CSF flow oscillations suggestive of vasomotion driven clearance of CSF and thereby of solutes and supportive of IPAD pathways of clearance (66).

### Neurofluid Physiology

To understand the interaction between the several space-competing compartments within the cranium, we must remind ourselves of the Monro–Kellie hypothesis, which remains a cardinal principle in the understanding of fluid movements (67). This hypothesis maintains that because the brain contents are enclosed in a non-expandable bony skull, the total brain volume must remain constant at all times to avoid a dangerous increase in ICP (68). However, with the recent discoveries of meningeal lymphatics and the understanding of mechanisms for brain waste clearance mechanisms, it has become necessary to revisit the original Monro–Kellie doctrine (69). With every systole, an increase in arterial pressure pumps ~700 ml of oxygenated blood, causing inflation of arteries, arterioles, and the microvascular bed (70). This expansion of vessels will squeeze ISF and CSF from the interstitium and promote flow. The creation of a pressure gradient between the cranial SAS and the spinal SAS will cause displacement of CSF toward the spinal SAS and facilitate venous outflow toward the extracranial neck vessels (3, 71). During diastole, as the elastic vessels relax, CSF flows back with little net forward displacement. Such pulsatile forces will also create a variable magnitude of brain tissue deformation, generating additional forces affecting blood flow, production, and absorption of ISF and CSF. The intrinsic viscoelasticity of the brain, or brain compliance, is the capacity of brain tissue to deform in conditions of intracranial pressure changes. Such mechanical and viscoelastic properties vary in different brain regions and depend on cellular morphology, capillary distribution, the compactness of white matter axons, their orientation, and ECM composition (72). These properties are different both at a macroscale (WM is stiffer than GM) and at a microscale (cortical GM is stiffer than hippocampal GM; WM in the corpus callosum is stiffer than WM in the corona radiata) (72). WM is three times stiffer than GM, accounting for differential response to compression load (73). Physiologic rheological properties of the brain can be measured *in vivo* by magnetic resonance elastography (74, 75). Thus, in one magnetic resonance elastography study, the compression of internal jugular veins in the neck was shown to increase CSF pulsatility in the brain and increase stiffness within the brain parenchyma in accordance with the Monro–Kellie doctrine (75).

### Cerebrovascular Damage and Neurodegeneration

Our attention is drawn to the intricate coupling of arterial, venous, CSF, and brain parenchymal dynamics; damage to any one of them can initiate a cascade of events affecting clearance of waste products in the brain and lead thereby to neurodegeneration. Reduced cerebral perfusion is considered a potential link between vascular risk factors and the development of SVD, vascular dementia, and Alzheimer's disease (AD) (76). The most important risk factors are advancing age and hypertension, both of which will hamper cerebral blood flow by directly damaging arterial walls and the microvasculature. Patients with SVD and AD often present with increased arterial stiffness, altered BBB permeability, VSMC loss, multiple fenestrations in the internal elastic lamina, remodeled arterial wall basement membranes, pericyte degeneration, increased intercapillary distance, reduced capillary density, increased arteriolar tortuosity, and swelling of astrocyte end feet, ultimately reducing the capacity for an optimal exchange of substances across the capillary endothelium (77–80). The inefficient transfer of pulsatile energy from the arterial bed toward the capillaries and the venous walls will disrupt hydrostatic forces. Arterial vasomotion will also be affected in several ways: direct arterial wall damage, deposition of amyloid-beta, and loss of cholinergic innervation of VSMCs. The geometry of ECS changes with age and disease, as free water within the parenchyma increases and toxic solutes such as amyloid-beta deposit within the extracellular space (81). In this scenario, the glymphatic/convective influx as well as IPAD will be hampered.

As the density of capillaries is lower in the white matter than in the gray matter and capillary basement membranes are the entry portals for IPAD by which ISF and solutes drain from brain tissue, the shortage of capillaries in the white matter may be a factor in a reduced capacity for IPAD in the white matter (82). Obstruction of CSF drainage from the cerebral ventricles results in dilatation of the ventricular system and the accumulation of fluid in the periventricular white matter in the acute stages of hydrocephalus with the slowly progressive destruction of white matter fibers and gliosis, suggesting that the capacity for IPAD is lower in the white matter compared with the gray matter (83).

Damage to veins, venules, and capillaries can also characterize other subtypes of SVD, such as perivenous collagenosis (84). This is characterized by concentric thickening of venular walls and pathological deposition of collagen resulting in leukoaraiosis or white matter hyperintensities on magnetic resonance imaging. Occlusion of venules and veins causes hypoperfusion and ischemia and affects the drainage of CSF via meningeal lymphatics (85).

There are several, albeit nonspecific, magnetic resonance imaging biomarkers such as dilated PVS, white matter hyperintensity, cerebral microbleeds, and superficial siderosis that characterize SVD, AD, and CAA that are an expression of impaired clearance of proteins and fluids, focal ischemia, and deposition of amyloid-beta within the walls of capillaries and neurodegeneration (82, 86–89). Neural tissue can become stiffer via several processes such as Wallerian degeneration, axonal atrophy, loss of oligodendroglial cells, microglial activation, neuroinflammation, and microvascular damage, resulting in a range of microstructural changes from increased tissue water content to progressive gliosis and loss of volume.

**Figure 2:**
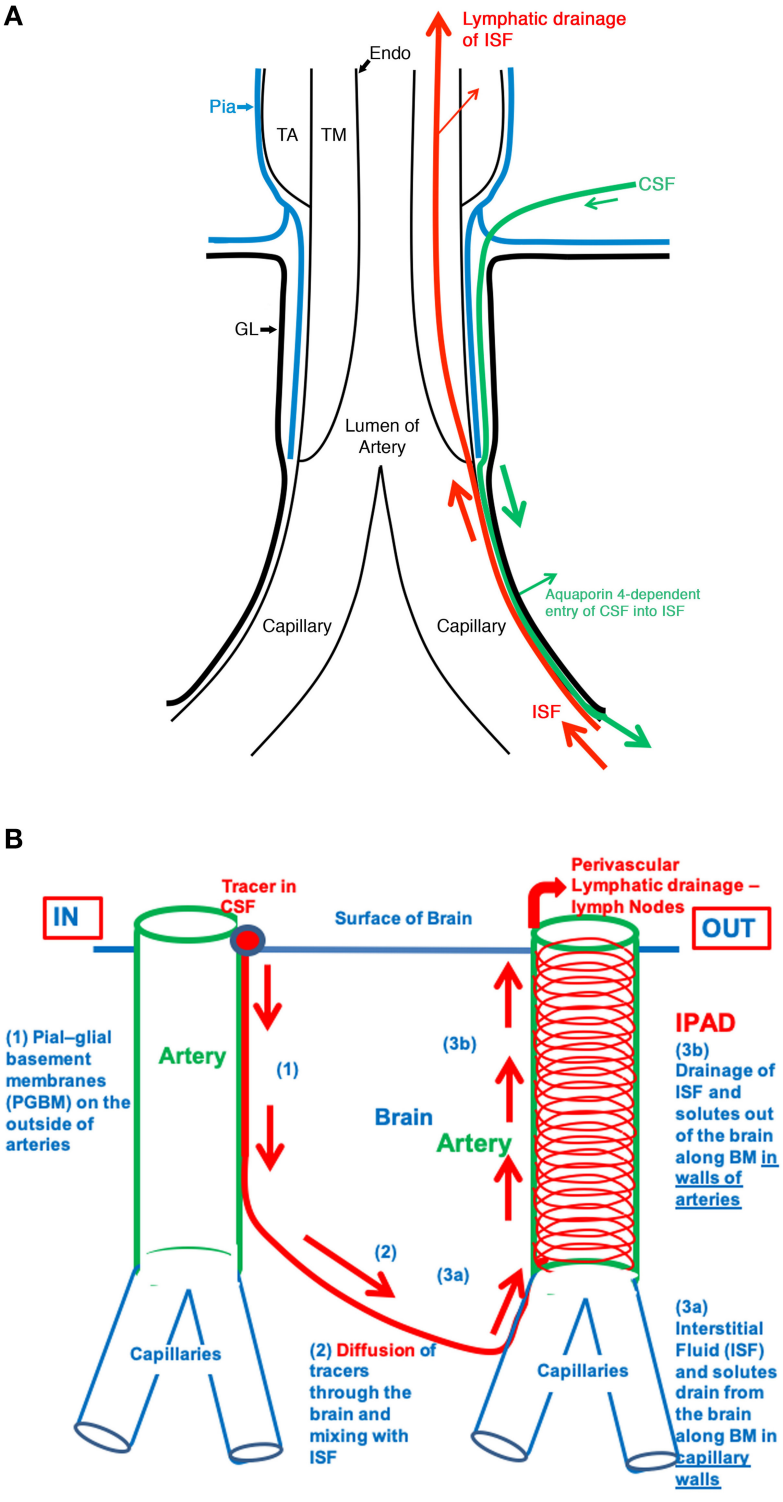
**(A)** The fine anatomy of the cerebral arterial wall. An artery is lined by endothelium (Endo) and coated by the tunica media (TM) composed of smooth muscle cells and by the outermost tunica adventitia (TA) composed of connective tissue. As it enters the brain, the artery loses the tunica adventitia but is still coated by a layer of pia-arachnoid (Pia) that intervenes between the artery and the glia limitans (GL) of the brain. As the arteriole divides into capillaries, the tunica media, and the layer of pia mater are lost. Thus, at the level of the capillary, the GL is in direct contact with the wall of the capillary. **(B)** Schematic representation of the IPAD and convective influx/glymphatic systems of the brain. On the left-hand side of the diagram, an artery enters the brain from the SAS, and an arteriole divides into capillaries. Tracers in the CSF enter the brain along the pial-glial basement membrane (1) between the pia mater and the GL (indicated by a green arrow) and enter the brain parenchyma and interstitial fluid by an aquaporin four-dependent mechanism, which is the glymphatic pathway (2). On the right-hand side of the diagram, the red arrows indicate the intramural perivascular lymphatic drainage pathway by which interstitial fluid (ISF) and solutes pass out of the brain along basement membranes in the walls of capillaries (3a) and along basement membranes surrounding smooth muscle cells in the tunica media of arterioles and arteries (3b). Reproduced with permission from Morris et al. (90)

There is substantial evidence that fluid movements in the brain are related such that damage to one compartment can lead to several events leading to neuroglial vascular compromise (Figure 2). In particular, the morphological damage to macro/microvasculature or their dysfunction will most likely compromise the movement of fluids, with impact on the perfusion of the brain and the drainage of CSF, ISF, altering the homeostasis of the brain, which in turn leads to neuronal cell loss and dementia.

## Author Contributions

NA wrote the manuscript. RC edited the manuscript. All authors contributed to the article and approved the submitted version.

## Conflict of Interest

The authors declare that the research was conducted in the absence of any commercial or financial relationships that could be construed as a potential conflict of interest.
